# Upregulation of xCT by KSHV-Encoded microRNAs Facilitates KSHV Dissemination and Persistence in an Environment of Oxidative Stress

**DOI:** 10.1371/journal.ppat.1000742

**Published:** 2010-01-29

**Authors:** Zhiqiang Qin, Eduardo Freitas, Roger Sullivan, Sarumathi Mohan, Rocky Bacelieri, Drake Branch, Margaret Romano, Patricia Kearney, Jim Oates, Karlie Plaisance, Rolf Renne, Johnan Kaleeba, Chris Parsons

**Affiliations:** 1 Department of Medicine, Hollings Cancer Center, Medical University of South Carolina, Charleston, South Carolina, United States of America; 2 Department of Craniofacial Biology, Hollings Cancer Center, Medical University of South Carolina, Charleston, South Carolina, United States of America; 3 Department of Dermatology, Hollings Cancer Center, Medical University of South Carolina, Charleston, South Carolina, United States of America; 4 Department of Pathology, Hollings Cancer Center, Medical University of South Carolina, Charleston, South Carolina, United States of America; 5 Medical Service, Ralph H. Johnson VA Medical Center, Charleston, South Carolina, United States of America; 6 Department of Molecular Genetics and Microbiology, Shands Cancer Center, University of Florida, Gainesville, Florida, United States of America; 7 Departments of Microbiology and Immunology and Molecular/Cell Biology, Uniformed Services University of the Health Sciences, F. Edward Herbert School of Medicine, Bethesda, Maryland, United States of America; University of Miami, United States of America

## Abstract

Upregulation of xCT, the inducible subunit of a membrane-bound amino acid transporter, replenishes intracellular glutathione stores to maintain cell viability in an environment of oxidative stress. xCT also serves as a fusion-entry receptor for the Kaposi's sarcoma-associated herpesvirus (KSHV), the causative agent of Kaposi's sarcoma (KS). Ongoing KSHV replication and infection of new cell targets is important for KS progression, but whether xCT regulation within the tumor microenvironment plays a role in KS pathogenesis has not been determined. Using gene transfer and whole virus infection experiments, we found that KSHV-encoded microRNAs (KSHV miRNAs) upregulate xCT expression by macrophages and endothelial cells, largely through miR-K12-11 suppression of BACH-1—a negative regulator of transcription recognizing antioxidant response elements within gene promoters. Correlative functional studies reveal that upregulation of xCT by KSHV miRNAs increases cell permissiveness for KSHV infection and protects infected cells from death induced by reactive nitrogen species (RNS). Interestingly, KSHV miRNAs simultaneously upregulate macrophage secretion of RNS, and biochemical inhibition of RNS secretion by macrophages significantly reduces their permissiveness for KSHV infection. The clinical relevance of these findings is supported by our demonstration of increased xCT expression within more advanced human KS tumors containing a larger number of KSHV-infected cells. Collectively, these data support a role for KSHV itself in promoting *de novo* KSHV infection and the survival of KSHV-infected, RNS-secreting cells in the tumor microenvironment through the induction of xCT.

## Author Summary

Herpesviruses are the most common etiologic agents of cancer in patients with suppressed immune function, and the Kaposi's sarcoma-associated herpesvirus (KSHV) is one of the most common causes of cancer in this setting. KSHV infection of new cell targets is critical for tumor progression, and a better understanding of how viral receptors on the surface of cells are regulated in the tumor microenvironment may lead to new therapies. KSHV encodes unique RNAs called microRNAs (KSHV miRNAs) that regulate a variety of cell functions. In this study, we show that KSHV miRNAs increase the susceptibility of cells to KSHV infection and protect infected cells from death induced by cancer-promoting reactive nitrogen species (RNS). They accomplish this in large part by increasing cell surface expression of a transport protein subunit called xCT. We also show that KSHV miRNAs increase secretion of RNS by infected cells, and that blocking RNS secretion reduces the ability of KSHV to infect cells. Therefore, by regulating xCT and RNS, we find KSHV is able to “fine-tune” cell function in order to maintain a stable population of infected cells which secrete cancer-promoting factors in the local environment. This work has important implications for developing new therapies to target xCT and reduce survival of KSHV-infected tumor cells.

## Introduction

Patients with immune deficiencies are at risk for life-threatening illnesses caused by herpesviruses, including the Kaposi's sarcoma-associated herpesvirus (KSHV). Bone marrow failure [1], lymphoproliferative syndromes [2],[3], and sarcoma [4] have all been etiologically linked to KSHV infection and occur with greater frequency in the setting of immune suppression related to HIV infection [5],[6] or organ transplantation [7],[8]. The most commonly encountered clinical manifestation of KSHV infection, Kaposi's sarcoma (KS), represents one of the most common tumors arising in the setting of HIV infection, one of the most common transplant-associated tumors, and a leading cause of morbidity and mortality [5]–[7]. Moreover, KS is the most common tumor arising in the general population in some geographic areas [9]. Despite the reduced incidence of KS in the modern era of highly active antiretroviral therapy (HAART) [10], KS is increasingly recognized in HIV-infected patients with suppressed HIV viral loads and elevated CD4^+^ T cell counts [11],[12]. Clinical responses to cytotoxic agents for systemic KS vary widely in published trials, and these agents incur many side effects which may exacerbate or add to those already incurred by antiretroviral or immunosuppressive agents [10],[13]. Given these shortcomings of existing therapies, novel targeted strategies are needed for the treatment or prevention of KS.

Published data support a role for KSHV-encoded genes in KS pathogenesis, including genes expressed primarily during lytic replication that facilitate angiogenesis and endothelial cell survival [14], and existing clinical data support this concept. An elevated KSHV viral load in the peripheral circulation predicts the onset and progression of both AIDS- and non-AIDS-related KS, and intralesional KSHV viral load correlates directly with tumor progression [15]–[17]. One retrospective clinical study demonstrated that ganciclovir, a nucleoside analog that inhibits viral DNA polymerase activity and reduces KSHV replication [18], reduced the incidence of KS in patients receiving organ transplants [19]. In addition, KS arising in the setting of well-controlled HIV infection may be explained in part by reduced KSHV-specific immunity despite general immune recovery with HAART [20],[21]. Together, these data suggest a role for ongoing KSHV replication and infection of naïve target cells in the progression of KS. Interestingly, neither KS lesional spindle cells nor cultured endothelial cells infected by KSHV *in vitro* efficiently maintain viral episomes when passed in culture [22],[23], suggesting the potential importance of additional microenvironmental factors within KS tumors for facilitating KSHV infection.

The amino acid membrane transport system x_c_^−^ consists of a conserved heavy chain, 4F2hc, and an inducible subunit, xCT, that mediates amino acid exchange [24]. x_c_^−^ exchanges intracellular glutamate for extracellular cystine at the cell membrane, and the latter is rapidly reduced in the intracellular space to cysteine and incorporated into glutathione (GSH) and other protein biosynthesis pathways [25]. This allows for restoration of intracellular GSH stores and protection of x_c_^−^-expressing cells from oxidative stress and cell death [25]. xCT expression is upregulated by physiological conditions that impact intracellular GSH levels, such as hypoxia, inflammation, and increased production of reactive species [25]. xCT was also recently identified as a fusion-entry receptor for KSHV and may mediate KSHV entry either in isolation or as part of a complex with other receptors for the virus [26],[27]. KSHV establishes infection within multiple xCT-expressing cell types that have been implicated in KS pathogenesis, including intralesional or circulating monocytes, intralesional macrophages, and endothelial cells [26]–[29]. However, whether KSHV itself also regulates xCT expression to promote viral infection of new cell targets or increase the longevity of KSHV-infected cells in the local environment is unknown.

miRNAs are small (19–23 nucleotides in length), non-coding RNAs that bind target mRNAs, marking them for degradation or post-transcriptional modification, and KSHV encodes 17 mature miRNAs which are expressed within KSHV-infected cells and KS lesions [30]–[33]. xCT expression is regulated through competitive binding of positive and negative transcription factors to an “Antioxidant Response Element” (ARE) in the xCT promoter [34],[35], and existing data suggest that negative transcription regulators of ARE may be targeted by KSHV miRNAs [36]–[40]. Therefore, using cell culture systems employing macrophages and endothelial cells, we sought to determine whether KSHV miRNAs regulate the expression of xCT, and if so, whether this infuences cell permissiveness for KSHV infection and protection of infected cells from oxidative stress.

## Results

### xCT is a principal determinant of macrophage permissiveness for KSHV infection

**Figure 1:**
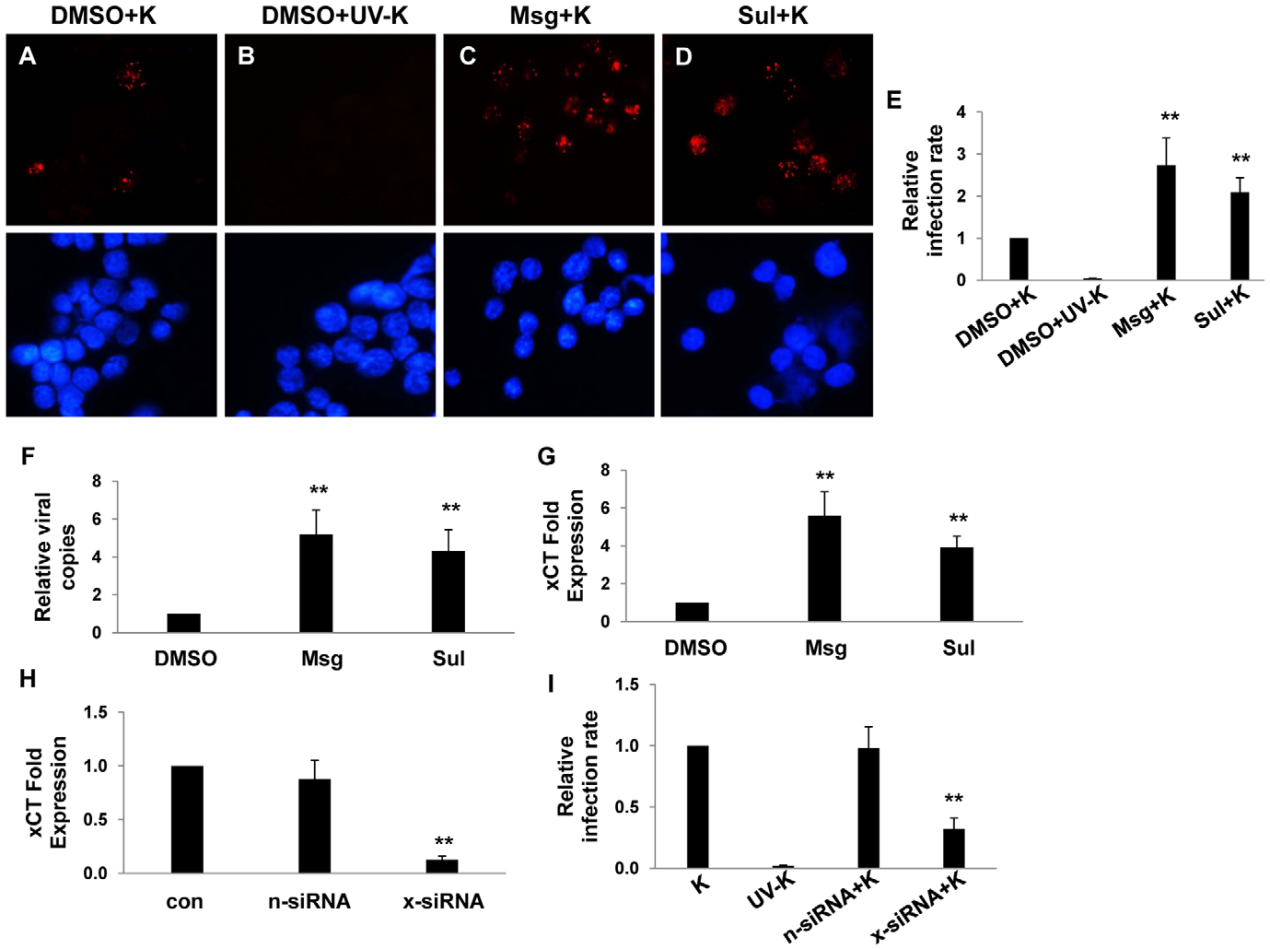
xCT mediates KSHV infection of macrophages. (**A**–**D**) 264.7 (“RAW”) cells were first incubated with Monosodium glutamate (Msg), Sulfasalazine (Sul) or vehicle control (DMSO) for 12 h followed by purified KSHV (K). 16 h later, IFA employing anti-LANA monoclonal antibodies and secondary antibodies conjugated to Texas Red were performed to identify expression of LANA signified by the typical punctate intranuclear expression pattern. Nuclei were identified using DAPI (blue). Some cells were incubated with UV-inactivated virus (UV-K) for negative controls. Representative images from one of three independent experiments are shown. (**E**) Relative infection rate was determined for groups in A–D as outlined in Methods. (**F**) qPCR was used to determine relative intracellular KSHV DNA content normalized to the vehicle control group (relative viral copies) as explained in Methods. (**G**) qRT-PCR was used to determine relative xCT transcript expression. (**H**) qRT-PCR was used to determine xCT transcript expression relative to control cells for cells transfected with either control (n) or xCT-specific siRNA. (**I**) Relative infection rates were calculated for groups in (H) using LANA IFA. For all assays, error bars represent the S.E.M. for three independent experiments. * * = p<0.01.

To first determine whether xCT expression correlates with macrophage susceptibility to KSHV infection, we utilized a BALB/c-derived murine macrophage cell line, 264.7 cells (“RAW” cells). We chose RAW cells given the recent demonstration of KSHV infection of murine macrophages *in vivo* [41], the identity (89%) and similarity (93%) of the murine xCT protein to its human counterpart [25], and the utility of these cells for gene transfer studies. xCT expression is induced indirectly by substrates that compete for cystine uptake by xCT, like monosodium glutamate (Msg) [42] and sulfasalazine (Sul) [43]. We found that Msg or Sul significantly increased the number of RAW cells expressing the KSHV latency-associated nuclear antigen (LANA) following their incubation with purified KSHV (Fig. 1A–E). This increase in the number of infected cells was reflected in an increase in viral episome copies for cells from Msg- and Sul-treated cultures (Fig. 1F), although IFA suggested that the number of episomes (LANA dots) per cell was unchanged (Fig. 1A–D). Msg and Sul also increased xCT transcript expression by RAW cells (Fig. 1G), and direct targeting of xCT with siRNA significantly reduced the number of LANA-positive cells following RAW cell incubation with KSHV (Fig. 1H and I). These data confirm the role of xCT as a principal determinant of RAW cell susceptibility to KSHV infection.

### KSHV miRNAs regulate xCT expression and macrophage permissiveness for KSHV infection

**Figure 2:**
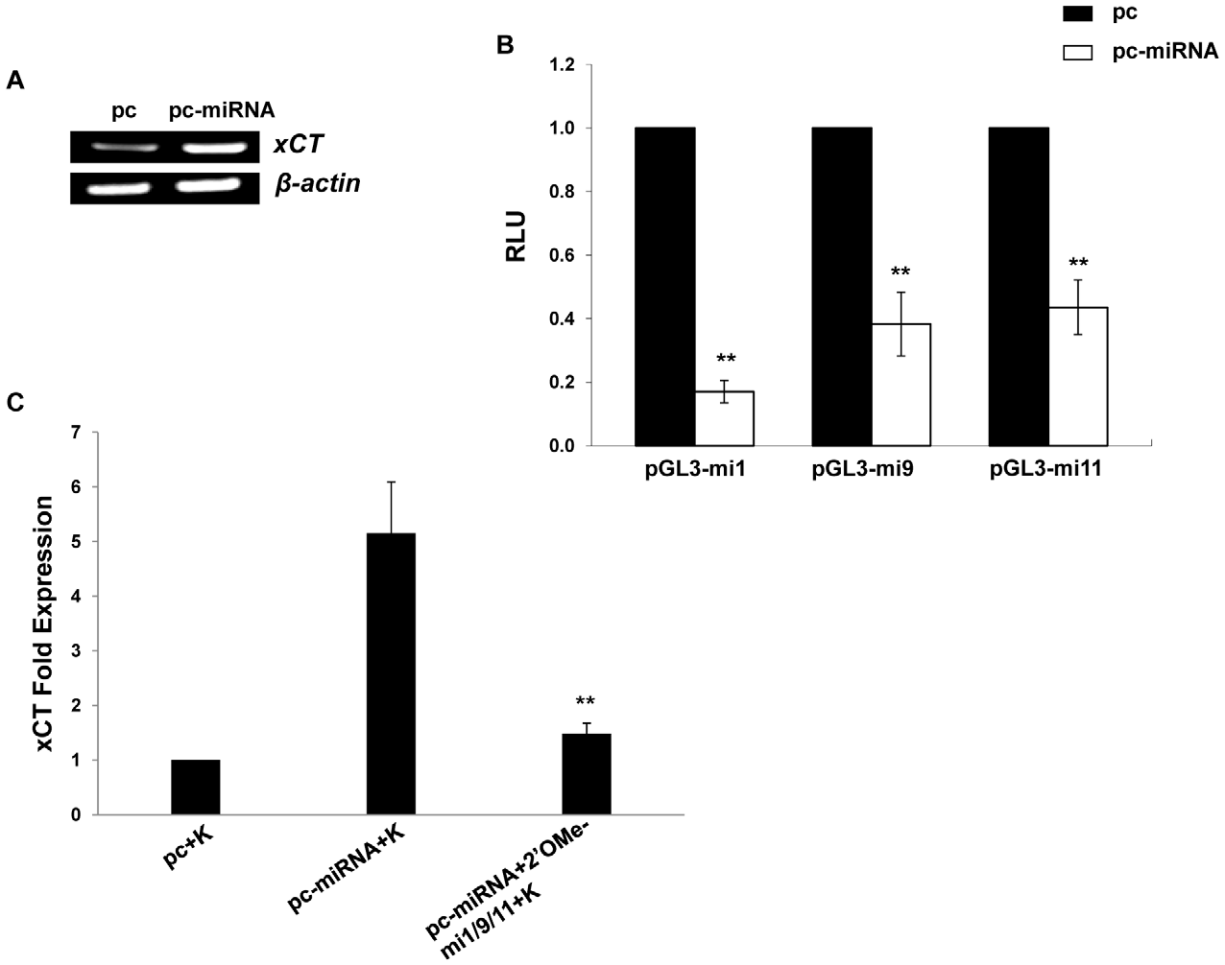
KSHV miRNAs upregulate xCT expression by macrophages. (**A**) RT-PCR was used to determine expression of xCT transcripts in RAW cells transfected with either control (pc) or miRNA-expressing vectors (pc-miRNA). β-actin was used as a loading control. (**B**) RAW cells were co-transfected with miRNA luciferase reporter constructs (pGL3-miX where X = complimentary sequence for the individual KSHV miRNAs noted) and either control or miRNA-expressing vectors. 48 h later, luciferase expression was determined for miRNA transfectants relative to controls (RLU). (**C**) Cells were transfected with control or miRNA-expressing vectors with or without 2'OMe RNA antagomirs targeting miR-K12-1, miR-K12-9, and miR-K12-11 (mi1/9/11). 48 h following subsequent incubation with KSHV (K), qRT-PCR was used to determine relative xCT transcript expression. For all assays, error bars represent the S.E.M. for three independent experiments. * * = p<0.01.

**Figure 3:**
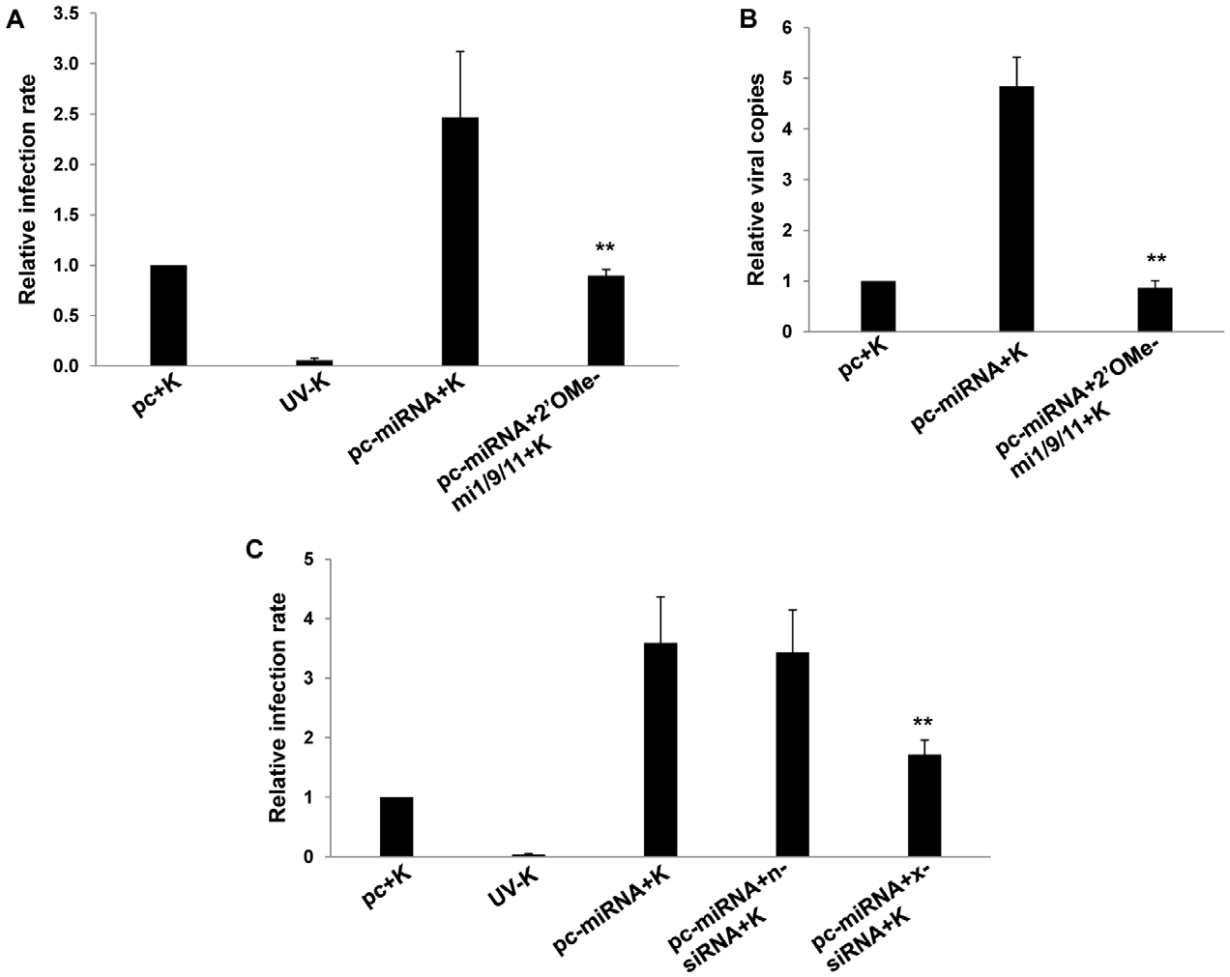
KSHV miRNA upregulation of xCT increases macrophage susceptibility to KSHV infection. (**A**) RAW cells were transfected with control or miRNA-expressing vectors then incubated with purified KSHV (K). LANA IFA were performed 16 h later, and relative infection rates were determined as outlined in Methods. (**B**) qPCR was used to determine relative intracellular KSHV DNA content for groups in A. (**C**) Cells were co-transfected with control or miRNA-expressing vectors and either control (n) or xCT-specific siRNA then incubated with purified KSHV. 16 h later, LANA IFA were used to determine relative infection rates. For all assays, error bars represent the S.E.M. for three independent experiments. * * = p<0.01.

To explore whether KSHV miRNAs regulate xCT expression, we co-transfected RAW cells with a construct encoding 10 of the 17 mature KSHV miRNAs described elsewhere [44]. Using semi-quantitative RT-PCR, we first confirmed upregulation of xCT with the collective expression of KSHV miRNAs encoded in the construct (Fig. 2A). Using a KSHV miRNA target prediction algorithm validated previously [36], we identified KSHV miRNA binding sites within 3'UTR of several murine genes associated with the regulation of xCT. The majority of binding sites were identified for 3 KSHV miRNAs: miR-K12-1, miR-K12-9, and miR-K12-11 (data not shown). To first determine whether these miRNAs were expressed within KSHV miRNA transfectants, we co-transfected cells with KSHV miRNAs and pGL3 luciferase reporter constructs encoding complimentary sequences for individual miRNAs (upon binding of pGL3 complimentary sequences to mature miRNAs, luciferase expression by pGL3 is repressed as shown elsewhere) [44]. This confirmed expression of miR-K12-1, miR-K12-9, and miR-K12-11 in these cells (Fig. 2B). Next, using qRT-PCR, we found that miR-K12-1, miR-K12-9, and miR-K12-11 are responsible for the upregulation of xCT in KSHV miRNA transfectants since targeting these 3 miRNAs with specific 2'OMe RNA antagomirs entirely suppressed this effect (Fig. 2C). Parallel experiments revealed that KSHV miRNAs increase intracellular KSHV viral load and viral transcript expression within macrophages following their incubation with KSHV (Fig. 3A and B). Once again, this effect was entirely suppressed by targeting miR-K12-1, miR-K12-9, and miR-K12-11 (Fig. 3A and B). In addition, we found that siRNA targeting of xCT significantly suppressed the KSHV miRNA-mediated increase in macrophage susceptibility to KSHV infection (Fig. 3C).

### KSHV miR-K12-11 suppresses BACH-1 to induce xCT expression and cell permissiveness for KSHV infection in macrophages and endothelial cells

**Figure 4:**
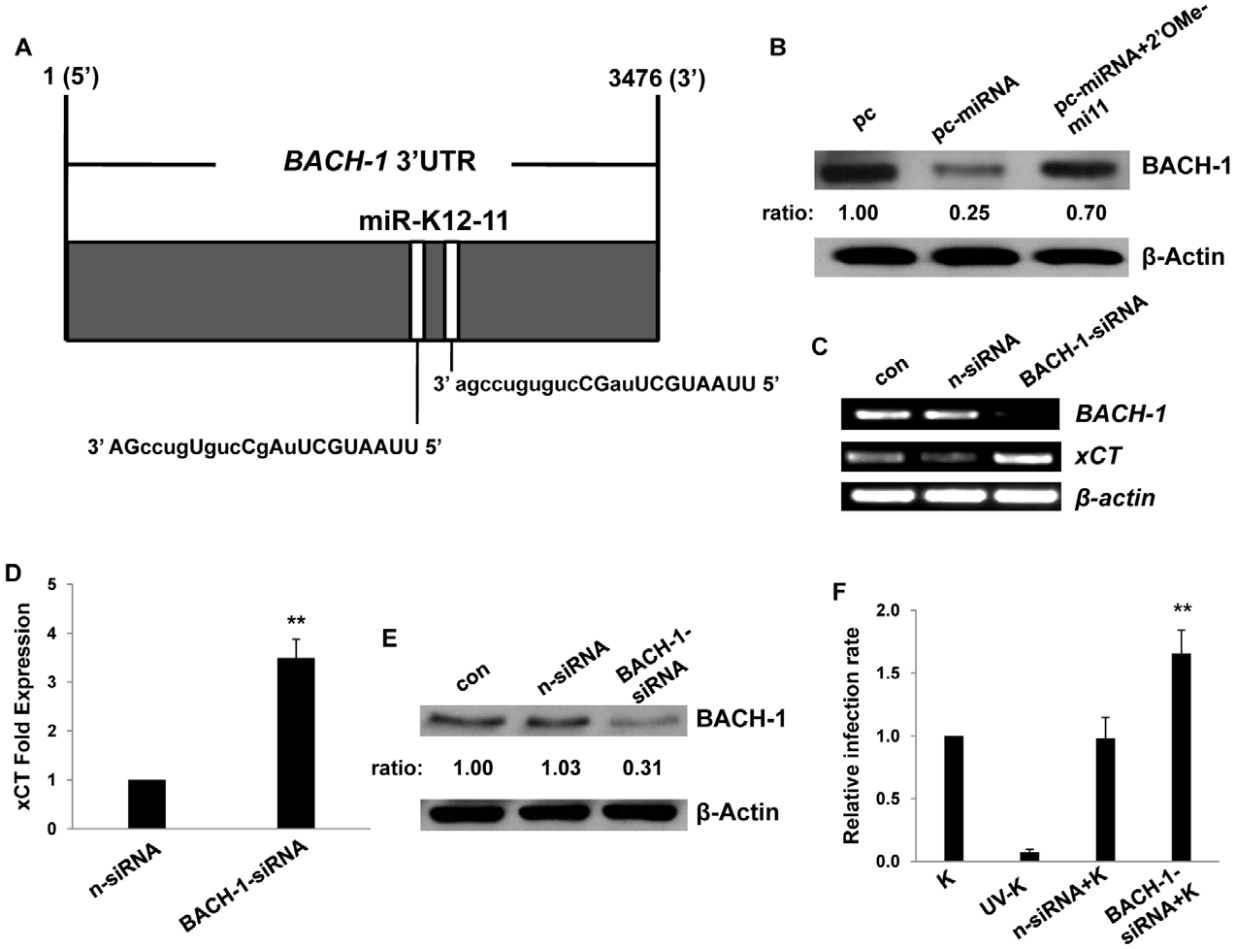
KSHV miRNAs upregulate xCT expression through repression of BACH-1. (**A**) Potential KSHV miRNA binding sites were identified within the 3'UTR of murine BACH-1 using an ad-hoc scanning program as described in Methods. miR-K12-11 nucleotides with matching base pairs depicted in capital letters bind within positions 2318–2339 and 2530–2551. (**B**) RAW cells were transfected with 1 µg control or miRNA-expressing vectors with or without 300 pmol of 2'OMe RNA antagomirs targeting miR-K12-11. 48 h later, BACH-1 expression was quantified by Western blot. β-Actin was used for loading controls. Numbers represent immunoreactivity relative to control transfectants as quantified using Image-J software. (**C–D**) RT-PCR (C) and qRT-PCR (D) were used to quantify transcripts for BACH-1 (C) and xCT (C and D), respectively, in controls cells or cells transfected with either control (n) or BACH-1-specific siRNA. (**E**) Western blots were used to quantify BACH-1 protein expression in siRNA-transfected cells for groups in (C). Immunoreactivity was quantified as in (B). (**F**) Cells were transfected with control or BACH-1 siRNA as above and subsequently incubated with KSHV. Relative infection rates were determined 12 h later using LANA IFA. Error bars represent the S.E.M. for three independent experiments. * * = p<0.01.

Our bioinformatics analyses revealed putative binding sites for miR-K12-11 within the 3'UTR of the murine BACH-1 gene (Fig. 4A). We subsequently confirmed KSHV miRNA suppression of BACH-1 within RAW cells, an effect largely reversed through direct targeting of KSHV miR-K12-11 (Fig. 4B). In addition, direct siRNA targeting of BACH-1 significantly increased basal levels of xCT expression in these cells (Fig. 4C–E) as well as macrophage permissiveness to KSHV infection (Fig. 4F), although to a lesser degree than the collective expression of miR-K12-1, miR-K12-9, and miR-K12-11 (Fig. 3).

**Figure 5:**
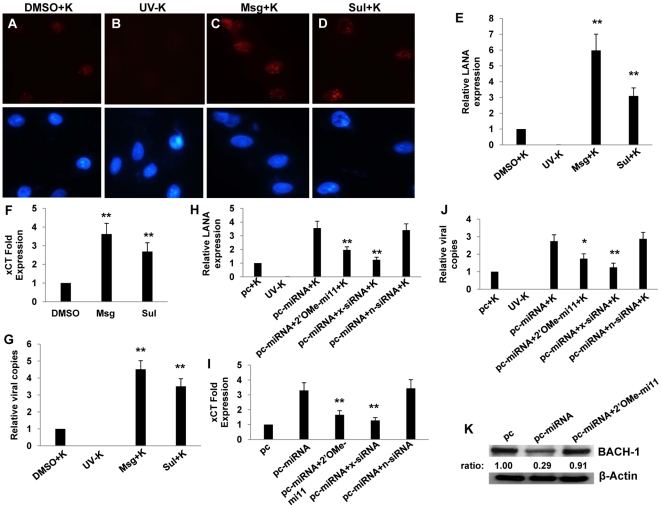
miR-K12-11 suppresses BACH-1 expression and increases endothelial cell susceptibility to KSHV through upregulation of xCT. (**A–G**) HUVEC were incubated with vehicle control (DMSO), Msg, or Sul for 12 h followed by purified KSHV (K) using an MOI∼0.5–1. 16 h later, LANA IFA were performed as previously described. (A–D) Representative images from one of three independent experiments are shown. (**H–K**) HUVEC were transfected with control or miRNA-expressing vectors along with a 2'OMe RNA antagomir for miR-K12-11 or either control (n) or xCT-specific siRNA. (**E**,**H**) Relative LANA expression was determined as described in Methods. (**F**,**I**) qRT-PCR was used to determine relative xCT transcript expression. (**G**,**J**) qPCR was used to determine relative intracellular KSHV DNA content normalized to controls. (**K**) Western blots were used to identify BACH-1 protein expression and immunoreactivity quantified as previously described. For all assays, error bars represent the S.E.M. for three independent experiments. * = p<0.05, * * = p<0.01 (For Fig. H–J, comparisons are relative to either pc-miRNA or pc-miRNA+K).

Published data have confirmed direct targeting of BACH-1 by miR-K12-11 in human cells [36]. To validate our observations and to determine their broader significance for human cells with known relevance to KS pathogenesis, we repeated our experiments using primary human umbilical vein endothelial cells (HUVEC). We found that Msg, Sul or KSHV miRNA transfection significantly increased xCT transcript expression and, based on IFA, KSHV episome copy number *per cell* following subsequent *de novo* infection (Fig. 5A–J). In contrast to what was observed for RAW cells, IFA indicated that the total number of infected HUVEC remained unchanged with these interventions (Fig. S1). In addition, either direct suppression of xCT by siRNA or concurrent inhibition of miR-K12-11 reduced xCT expression and intracellular viral load in KSHV miRNA transfectants (Fig. 5H–J). Collective expression of KSHV miRNAs also reduced BACH-1 expression in HUVEC, an effect suppressed with concurrent inhibition of miR-K12-11 (Fig. 5K).

### KSHV miRNAs upregulate macrophage secretion of reactive nitrogen species (RNS) and protect macrophages from RNS-induced cell death

**Figure 6:**
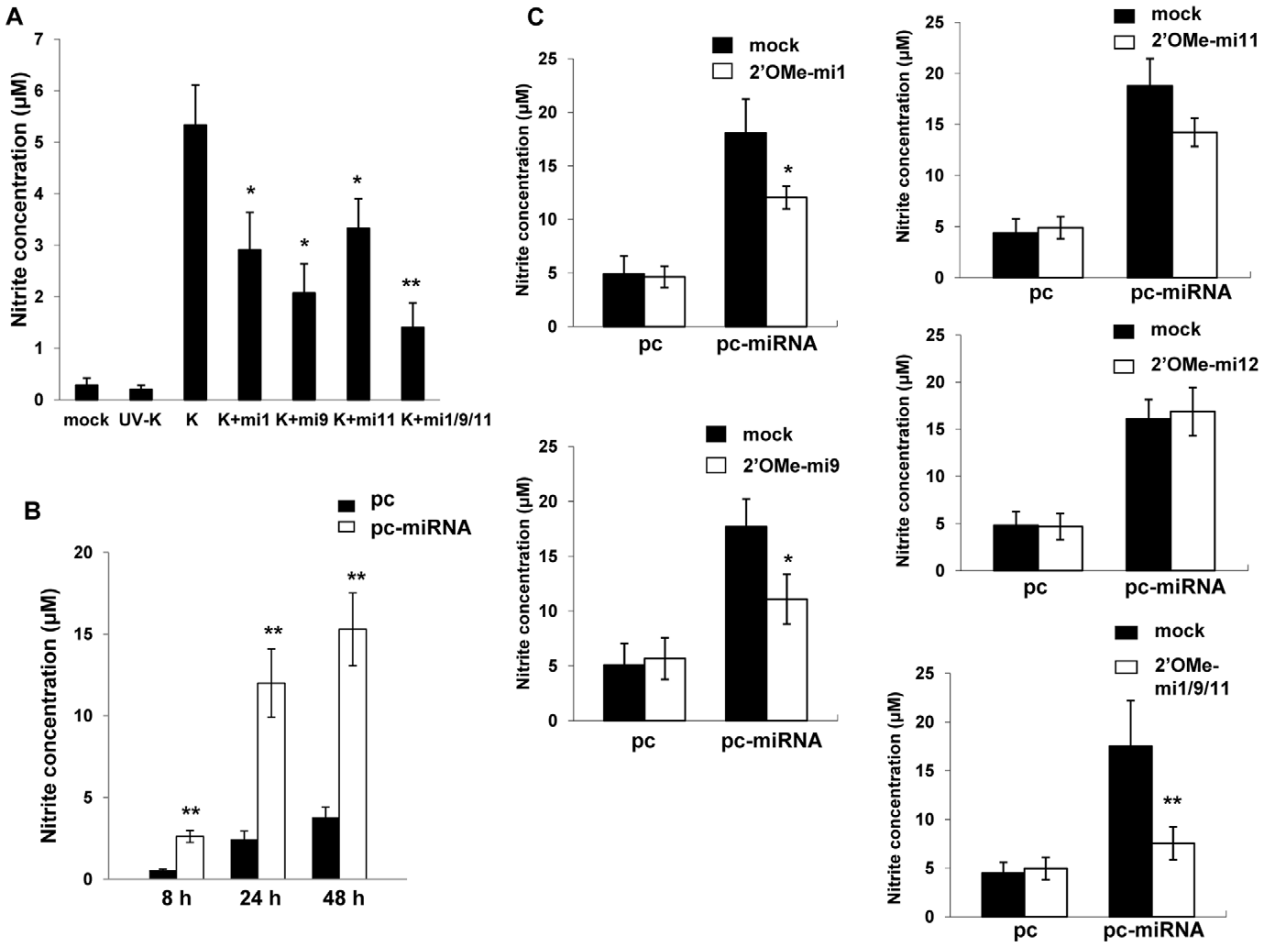
KSHV miRNAs induce reactive nitrogen species (RNS) secretion by macrophages. (**A**) RAW cells were transfected with 2'OMe RNA antagomirs targeting miR-K12-1, miR-K12-9, and miR-K12-11 or all three together (mi1/9/11). Cells were subsequently incubated with purified KSHV (K) and nitrite quantified within culture supernatants as described in Methods. (**B**) Cells were transfected with control or miRNA-expressing vectors and nitrite quantified within culture supernatants at the times indicated. (**C**) Cells were co-transfected with either control or miRNA-expression constructs without (mock) or with specific 2'OMe RNA antagomirs. As an additional control, some cells were transfected with an antagomir targeting miR-K12-12 which is not expressed by the miRNA-expressing construct. For all assays, error bars represent the S.E.M. for three independent experiments. * = p<0.05, * * = p<0.01.

xCT restoration of intracellular glutathione and increased scavenging of free radicals reduces cell death resulting from nitration of proteins, lipids, and nucleic acids by RNS [25],[45],[46]. Because we observed increased xCT expression induced by KSHV miRNAs, we hypothesized that KSHV may also increase RNS secretion by macrophages and that xCT upregulation would serve as an auto-protective mechanism in this environment. Using a standard Greiss reaction assay for quantifying nitrite in culture supernatants as a surrogate measure of RNS secretion, we found that KSHV infection of RAW cells induced a ∼20-fold increase in RNS secretion and that the majority of this effect was mediated through the collective expression of miR-K12-1, miR-K12-9, and miR-K12-11 (Fig. 6A). A similar pattern was observed following overexpression of KSHV miRNAs (Fig. 6B and C). Non-specific TLR activation, as might be initiated by mature miRNAs or their precursors, is capable of inducing RNS production [47],[48]. However, specific inhibitors of MyD88-independent and -dependent toll-like receptor (TLR) pathways failed to reduce induction of RNS secretion by KSHV miRNAs, suggesting that this effect is not mediated through TLR activation (Fig. S2).

**Figure 7:**
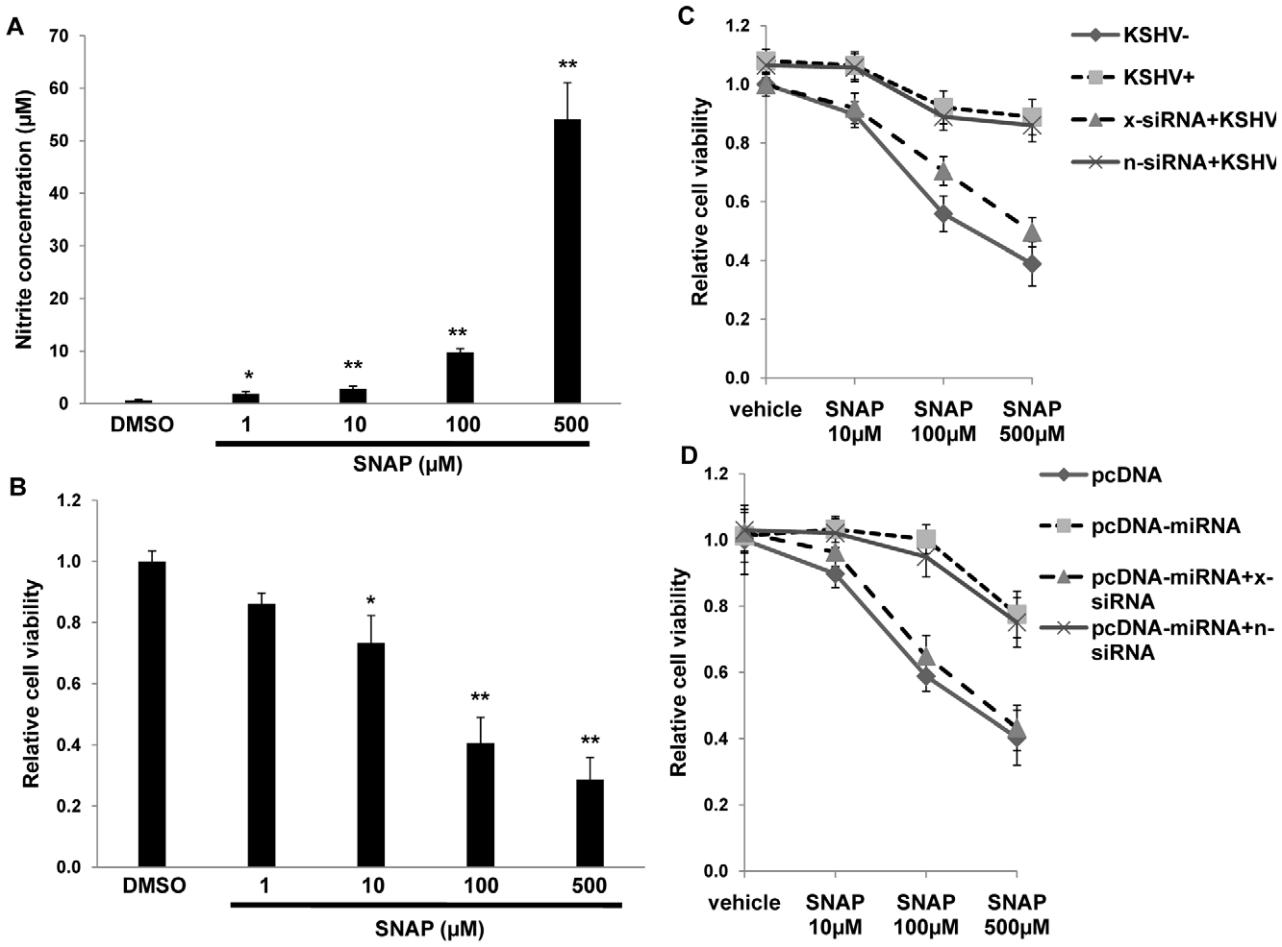
KSHV miRNAs enhance macrophage survival in an environment of oxidative stress through the upregulation of xCT. (**A**) RAW cells were treated with the indicated concentrations of SNAP or vehicle control for 12 h prior to nitrite quantification within culture supernatants. (**B**) Relative cell viability was determined for groups in (A) as described in Methods. (**C**) Cells were transfected with either control (n) or xCT-specific (x) siRNA and incubated with UV-K (KSHV−) or KSHV (KSHV+) 48 h later. (**D**) Cells were co-transfected with xCT siRNA and either control or miRNA-expressing vectors for 48 h, then incubated for an additional 12 h with SNAP prior to viability determinations. For all assays, error bars represent the S.E.M. for three independent experiments. * = p<0.05, * * = p<0.01.

To determine whether upregulation of xCT by KSHV miRNAs offers a protective mechanism for macrophages in an environment rich in RNS, we first established that provision of the nitric oxide (NO) donor S-nitroso-N-acetylpenicillamine (SNAP) [49] increased RNS concentrations within RAW cell culture supernatants and induced cell death in a dose-dependent manner (Fig. 7A and B). Subsequently, we found that either KSHV infection or overexpression of KSHV miRNAs significantly increased macrophage resistance to SNAP-induced cell death. Moreover, siRNA experiments confirmed that this effect was mediated primarily through the upregulation of xCT (Figs. 7C and D).

### RNS facilitate KSHV infection of macrophages

**Figure 8:**
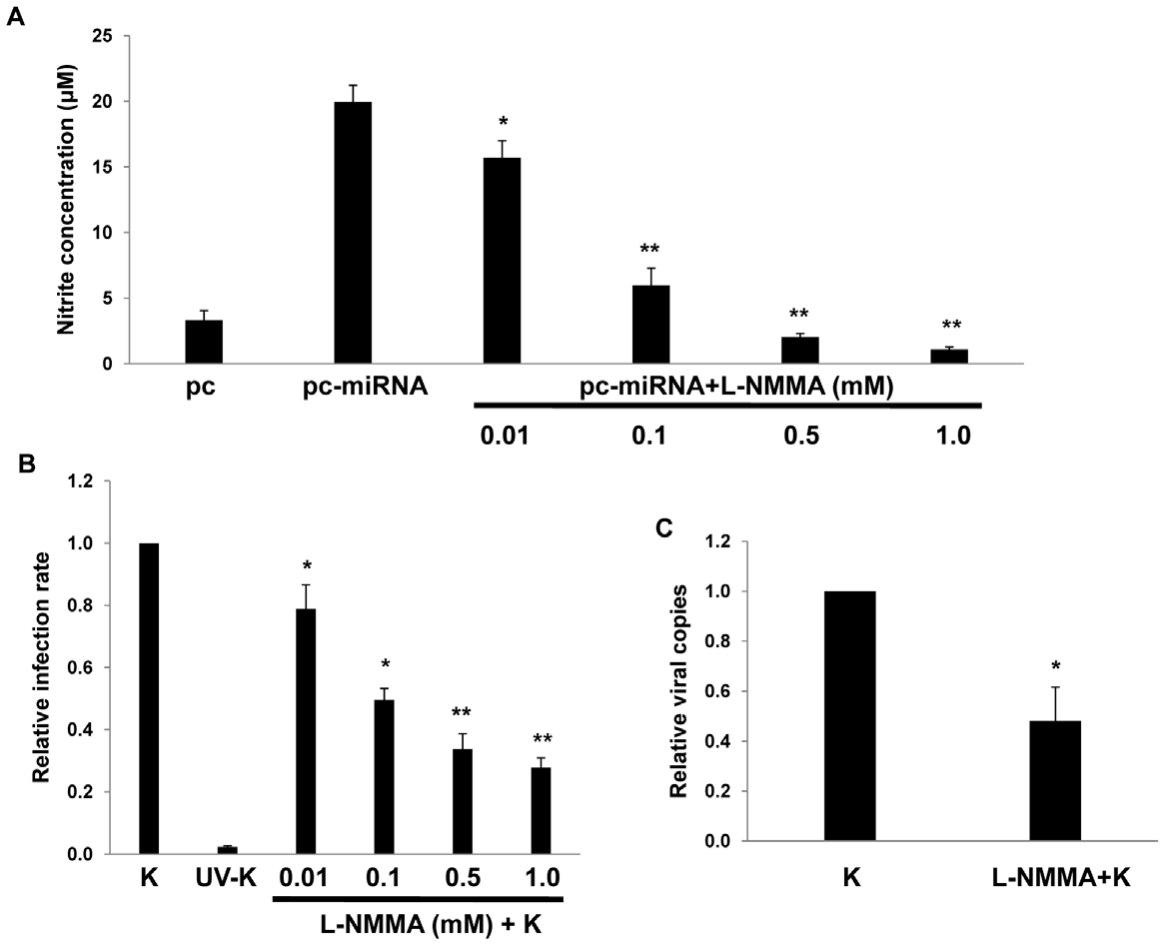
RNS inhibition reduces macrophage susceptibility to KSHV infection. (**A**) RAW cells were first transfected with 1 µg of either control or miRNA-expressing vectors then incubated with L-NMMA for 12 h prior to nitrite quantification in culture supernatants. (**B**) Cells were incubated with L-NMMA for 12 h prior to their incubation with purified KSHV (K) for 2 h. Following an additional 12 h, LANA IFA were performed and relative infection rates determined as described in Methods. (**C**) qPCR was used to determine relative intracellular KSHV DNA content for KSHV-infected cells pre-treated with either vehicle or 0.5mM L-NMMA. In all assays, error bars represent the S.E.M. for three independent experiments. * = p<0.05, * * = p<0.01.

RNS are expressed within KS lesions [50], but whether RNS themselves influence *de novo* KSHV infection is unknown. To reduce macrophage secretion of RNS, we incubated RAW cells with L-N^6^-monomethyl-arginine (L-NMMA), an inhibitor of all forms of nitric oxide synthase (NOS) [51] that induces no discernable toxicity for RAW cells over a wide range of concentrations (Fig. S3). Interestingly, L-NMMA significantly reduced secretion of RNS initiated by KSHV miRNAs and reduced *de novo* KSHV infection of macrophages in a dose-dependent manner (Fig. 8), suggesting a role for NOS and RNS in facilitating *de novo* KSHV infection.

### xCT expression is increased within more advanced KS lesions

**Figure 9:**
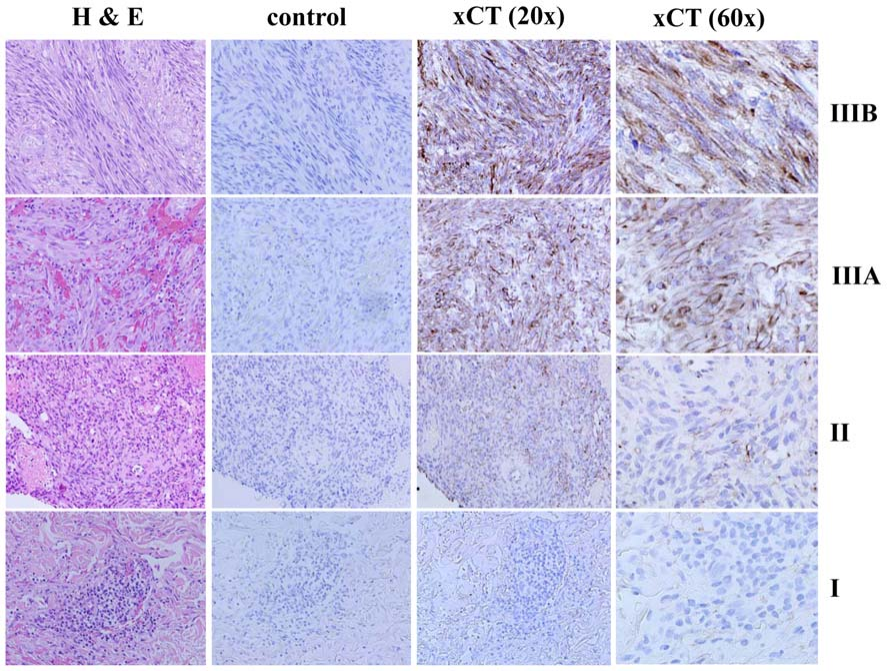
xCT expression within KS lesions correlates with tumor stage. KS diagnosis and histopathologic staging were independently confirmed by a dermatopathologist using hematoxylin and eosin (H & E). Tumors were then processed for immunohistochemistry as described in Methods using either pre-immune sera (control) or xCT anti-sera. xCT expression is revealed by dark brown membrane-associated staining in contrast to blue nuclear staining. Representative images from all stages (I = patch, II = plaque, III = nodule) are shown, including two different stage III tumors (A and B). All images are shown at original magnification ×20 or 60.

KSHV miRNAs are expressed within KS lesions [30]–[32] but to our knowledge, expression of xCT within KS tissue has never been demonstrated. To address this, we used immunohistochemistry to quantify xCT expression within KS skin lesions representing the full spectrum of histopathologic progression of KS. We found that stage I tumors (patches) and stage II tumors (plaques) exhibited either no or minimally discernable xCT expression, respectively (Fig. 9). In contrast, stage III tumors (nodules) exhibited easily discernable membrane expression of xCT by the majority of cells in these lesions, including nearly all spindle-shaped cells (Fig. 9). Moreover, we confirmed that stage III tumors exhibited significantly more LANA^+^ cells than stage I lesions (Fig. S4) in agreement with published data [52],[53] as well as our observed correlation between KSHV viral load and xCT expression in our *in vitro* experiments.

## Discussion

In this study, we found that KSHV-encoded miR-K12-11 upregulates the expression of xCT in macrophages and endothelial cells, in part through suppression of a negative regulator of gene transcription, BACH-1. We also found that KSHV miRNAs induce macrophage secretion of RNS and protect these cells from RNS-induced cell death through the upregulation of xCT. Moreover, reducing NOS activity and RNS secretion by macrophages reduces their permissiveness for KSHV. Finally, we found that cells within more advanced human KS tumors express more xCT than cells from early-stage lesions. We hypothesize, therefore, that KSHV miRNAs facilitate KS pathogenesis through cooperative mechanisms that regulate xCT and RNS secretion to promote ongoing *de novo* infection and survival of infected cells in the tumor microenvironment.

### Implications of the regulation of BACH-1, xCT expression, and cell susceptibility to KSHV infection by KSHV miRNAs

xCT expression is differentially regulated during oxidative stress through transcription factor binding to the *cis*-acting ARE in its promoter [34],[35]. Transcription factors that bind to the ARE include a positive regulator known as Nuclear factor erythroid 2-related factor-2 (Nrf-2) [35] and negative regulators, including BACH-1 and c-Maf, which competitively reduce Nrf-2 binding to the ARE thereby repressing ARE-mediated gene expression [37],[38]. KSHV miRNAs are expressed within KSHV-infected cells and KS lesions [30]–[32], and existing data suggest that both BACH-1 and c-Maf are targeted by KSHV miRNAs [36],[39],[40]. More specifically, KSHV miR-K12-11, an ortholog of cellular miR-155, targets and reduces expression of BACH-1 [36]. miR-155 downregulates c-Maf expression by T cells [40], and KSHV miRNAs downregulate c-Maf expression in endothelial cells [39]. Therefore, we hypothesized that miR-K12-11, in cooperation with other KSHV miRNAs, regulates xCT expression.

We found that miR-K12-11 downregulated BACH-1 and induced xCT expression in both macrophages and endothelial cells, although additional experiments using site-directed mutagenesis are needed to confirm direct interactions between BACH-1 and the xCT ARE in murine cells. Further validating our findings, we found that BACH-1 targeting by siRNA increased macrophage permissiveness for infection by approximately 70% (Fig. 4F), although overexpression of multiple miRNAs increased permissiveness by approximately 250% (Fig. 3E). Differences in transfection efficiency for siRNA and the KSHV miRNA constructs could be partially responsible for this discrepancy, but we hypothesize that it is due in part to the effect of multiple KSHV miRNAs, including miR-K12-1 and miR-K12-9, and the cooperative targeting of multiple genes. Our initial screen for KSHV miRNA binding sites within murine and human genes known to regulate xCT expression and RNS secretion revealed multiple binding sites for miR-K12-1, miR-K12-9 and miR-K12-11 (data not shown). These analyses also revealed binding sites for miR-K12-4, although not miR-K12-1 or miR-K12-9, within both murine and human BACH-1 3'UTR sequences (not shown), although we have not yet confirmed the functional impact of miR-K12-4 expression on BACH-1 or xCT expression. Additional studies are needed to confirm direct targeting of BACH-1 or other genes by these KSHV miRNAs and to characterize the functional impact of this targeting for expression of xCT and other ARE-containing genes regulated by BACH-1, including those involved in the generation of RNS (see below).

To our knowledge, these data are the first to suggest a role for a herpesvirus in the autocrine upregulation of its own receptor, although whether increased cell permissiveness for KSHV entry following initial infection and miRNA expression is “accidental” or “purposeful” in the context of KSHV-host evolution remains debatable. In addition to promoting cell survival (see below), autocrine upregulation of xCT may provide evolutionary advantages for the virus achieved through an increase in intracellular viral load that were not addressed by our studies. This concept is supported by several reports revealing that a significant proportion of KSHV-infected tumor cells, including those within KS lesions, contain multiple viral clones [54]–[56]. Another study showed that the downregulation of MHC Class I (MHC-I) in KSHV-infected cells is directly proportional to the level of expression of the KSHV modulator of immune recognition 2 (MIR2) and intracellular KSHV episome copy number [57], implying that increasing intracellular viral copies may promote reduced KSHV epitope presentation to CD8^+^ T cells as a mechanism for immune evasion. Our IFA indicated that for RAW cells, miRNA upregulation of xCT increased the permissiveness of uninfected cells for KSHV, although not viral episome copies within individual cells. In contrast, miRNA upregulation of xCT increased HUVEC viral episome copies per cell following subsequent infection, although not the total number of infected cells. Our studies did not directly address whether the observed increase in episome copies per cell for HUVEC is the result of intracellular episome replication or “superinfection” with exogenous virions. Experiments utilizing limiting dilution PCR [58] or single cell imaging techniques [41],[59] could be used to confirm whether intracellular KSHV viral load and miRNA expression correlate with xCT expression on a single cell level and to address the possibility that KSHV miRNA upregulation of xCT increases cell permissiveness for subsequent virion entry. It is interesting to speculate whether autocrine regulation of surface receptors by KSHV miRNAs differs depending on the cell type, and whether soluble factors released by infected cells differentially influence xCT expression for different cell types.

### Implications of the regulation of RNS secretion, xCT expression, and survival of KSHV-infected cells by KSHV miRNAs

Our data indicate a role for KSHV miRNAs in the induction of RNS secretion and the protection of cells from RNS-induced cell death through the upregulation of xCT. Of additional relevance, we found that L-NMMA, an inhibitor of NOS, reduced KSHV miRNA-induced secretion of RNS and *de novo* KSHV infection. Additional studies are currently underway to elucidate the mechanism for these observations. Through the nitration of either extracellular or intracellular proteins, RNS activate Nrf-2 [34],[35],[60] and, therefore, may upregulate xCT expression through both autocrine and paracrine mechanisms. It is also conceivable that miR-K12-11 downregulation of BACH-1 increases expression of other ARE-containing genes involved in the induction of RNS or the protection of cells from oxidative stress, including heme oxygenase-1 (HO-1) [34],[35]. Interestingly, HO-1 is expressed within KS lesions, and KSHV infection of endothelial cells induces activation of HO-1 [61]. It is probable that RNS secretion and downstream consequences are mediated through the collective targeting of multiple genes by KSHV miRNAs, and that this may occur within a variety of KSHV-infected cells with the capacity to secrete RNS, including endothelial cells and dendritic cells [25]. Characterization of miRNA regulation of RNS for a broader array of cell types relevant to KS pathogenesis is underway. Furthermore, our studies do not address whether KSHV miRNAs regulate secretion of reactive oxygen species (ROS) by infected cells, and it is conceivable that ROS play a role in the paracrine regulation of xCT or other events pertaining to KSHV infection. Studies are ongoing in our laboratory to define the relative importance of specific reactive species in the regulation of xCT expression and KSHV dissemination in the microenvironment.

Multiple studies implicate RNS in KS pathogenesis [46], [50], [62]–[66]. RNS and NOS are expressed within KS lesions [50],[62], and RNS induce endothelial cell migration, proliferation and angiogenesis [46] as well as T cell apoptosis [63]. Moreover, existing data support a role for KSHV in the regulation of superoxide dismutase (SOD) in the KS microenvironment [50],[67], and cytokines associated with KS pathogenesis have been implicated in the activation of RNS secretion by macrophages [64],[65]. Interestingly, a recent publication demonstrated that Rac1 transgenic mice overexpressing NADPH-oxidase-dependent reactive species developed KS-like lesions and that systemic administration of the antioxidant N-acetylcysteine reduced KS formation in this model [66]. Preliminary experiments performed in our laboratory have revealed that inhibition of NADPH-oxidase using diphenylene iodonium (DPI) also reduces KSHV miRNA-induced RNS secretion and infection of naïve cells (data not shown). In addition, at least one study has implicated cellular miRNAs in the regulation of Rac1 [68]. Therefore, our findings have important implications for paracrine regulation of cellular events pertaining to KS pathogenesis, and systemic inhibition of RNS may interfere with many of these events including viral dissemination and angiogenesis.

### Clinical implications of xCT expression within KS lesions

We have demonstrated that xCT is expressed to a greater extent within more advanced KS lesions containing a greater number of KSHV-infected cells. To our knowledge, these are the first data to demonstrate xCT expression in clinical samples from KSHV-infected patients and are consistent with published data documenting higher KSHV intratumoral viral loads within more advanced KS lesions [52],[53]. Importantly, they also support our hypothesis that KSHV upregulation of xCT facilitates expansion of the KSHV reservoir in the microenvironment and KS progression. Moreover, our observation that spindle-shaped cells within stage III tumors express xCT is consistent with our data revealing upregulation of xCT and KSHV permissiveness for endothelial cells *in vitro* by KSHV miRNAs. Additional studies are needed to confirm whether KSHV miRNAs, BACH-1 and other putative xCT regulatory factors are differentially expressed during different stages of KS progression. Future translational studies may also shed light on whether quantifying xCT in clinical samples provides additional prognostic information for patients at risk for KS, and whether targeting xCT or its regulatory pathways will offer a useful approach for the treatment or prevention of this disease.

## Materials and Methods

### Cell culture

BCBL-1 cells were grown in RPMI 1640 media (Gibco) supplemented with 10% fetal bovine serum (FBS), 10 mM HEPES (pH 7.5), 100 U/mL penicillin, 100 µg/mL streptomycin, 2 mM L-glutamine, 0.05 mM β-mercaptoethanol, and 0.02% (wt/vol) sodium bicarbonate. Murine macrophages, RAW 264.7 cells (RAW cells), were obtained from American Type Culture Collection (ATCC) and grown in Dulbecco's modified Eagle's medium (DMEM, Gibco) supplemented with 10% FBS. HeLa cells were grown in DMEM supplemented with 10% FBS, 100 U/mL penicillin and 100 µg/mL streptomycin. Human umbilical vein endothelial cells (HUVEC) were grown in DMEM/F-12 50/50 medium (Cellgro) supplemented with 5% FBS and 0.001 mg/mL Puromycin (Sigma).

### Antibodies and reagents

Antibodies recognizing BACH-1 (H-130) and β-Actin were purchased from Santa Cruz Biotechnology (Santa Cruz, CA) and Sigma (St. Louis, MO), respectively. Msg, Sul and L-NMMA were purchased from Sigma (St. Louis, MO). SNAP was purchased from Invitrogen (Eugene, Oregon).

### Transfection assays

A 2.8 Kbp construct encoding 10 individual KSHV microRNAs (pcDNA-miRNA, containing miR-K12-1/2/3/4/5/6/7/8/9/11), and luciferase reporter constructs encoding complimentary sequences for individual miRNA (pGL3-miRNA sensors), have been validated previously in transfection assays for expression of KSHV miRNAs [44]. These constructs were used to transiently transfect RAW cells and HUVEC. For inhibition of mature miRNAs, 2'OMe RNA antagomirs were designed and purchased from Dharmacon (Chicago, IL) as previously described [44]. BACH-1, xCT, and non-target (control) siRNAs (ON-TARGET plus SMART pool) were also purchased from Dharmacon. Cells were transfected with 1 µg pcDNA-miRNA, 0.5 µg pGL3-miRNA sensors, 300 pmol 2'OMe RNA antagomirs, siRNAs, and/or 1 µg pcDNA for negative controls in 12-well plates using Lipofectamine 2000 (Invitrogen, Carlsbad, CA) and/or DharmaFECT Transfection Reagent (Dharmacon, Chicago, IL) for 48 h prior to their incubation with KSHV. For miRNA inhibitor assays, control cells were transfected with a 2'OMe RNA antagomir targeting miR-K12-12, a KSHV miRNA not encoded by the pcDNA-miRNA construct. For luciferase expression assays, cells were incubated with 100 µL lysis buffer (Promega, Madison, WI), and luciferase activity determined within lysates using a Berthold FB12 luminometer (Titertek, Huntsville, AL). Light units were normalized to total protein levels for each sample using the BCA protein assay kit according to the manufacturer's instructions (Pierce, Rockford, IL). Transfection efficiency was assessed through co-transfection of a lacZ reporter construct kindly provided by Dr. Yusuf Hannun (Medical University of South Carolina, Charleston, SC), and β-galactosidase activity determined using a commercially available β-galactosidase enzyme assay system according to the manufacturer's instructions (Promega, Madison, WI). 3 independent transfections were performed for each experiment, and all samples were analyzed in triplicate for each transfection.

### Nitrite quantification

Nitrite concentrations within culture supernatants were determined using the Griess Reagent System (Promega, Madison, WI) according to the manufacturer's instructions.

### Immunoblotting

Cells were lysed in buffer containing 20 mM Tris (pH 7.5), 150 mM NaCl, 1% NP40, 1 mM EDTA, 5 mM NaF and 5 mM Na_3_VO_4_. 30 µg of total cell lysate was resolved by SDS–10% PAGE and transferred to nitrocellulose membranes prior to incubation with antibodies for proteins of interest as well as β-Actin for loading controls. Immunoreactive bands were developed by enhanced chemiluminescence reaction (Perkin-Elmer), visualized by autoradiography, and quantified using Image-J software.

### Bioinformatics analysis

The 3'UTR sequences of BACH-1 and other RNS-associated genes were obtained from Ensembl (http://www.ensembl.org). 3'UTRs were analyzed to extract all potential KSHV miRNA binding sites using an ad-hoc scanning program specifically developed to assess 3'UTR KSHV miRNA seed sequence matching, as validated previously [36].

### KSHV purification and infection

BCBL-1 cells were incubated with 0.6 mM valproic acid for 4–6 days, and KSHV was purified from culture supernatants by ultracentrifugation at 20,000×g for 3 h, 4°C. The viral pellet was resuspended in 1/100 the original volume in the appropriate culture media, and aliquots were frozen at −80°C. Target cells were incubated with concentrated virus in the presence of 8 µg/mL polybrene (Sigma-Aldrich) for 2 h at 37°C. Inactivated KSHV used for negative controls was prepared by incubating viral stocks with ultraviolet (UV) light (1200 J/cm^2^) for 10′ in a CL-1000 Ultraviolet Crosslinker (UVP). The concentration of infectious viral particles used in each experiment (multiplicity of infection [MOI]) was calculated as previously described [57],[69].

### Immunofluorescence assays and determination of relative infection rates

1×10^4^ RAW cells or HUVEC were seeded per well in eight-well chamber slides (Thermo Fisher, Rochester, NY) and incubated with viral stocks (MOI∼10 for RAW cells, MOI∼0.1–1 for HUVEC) in the presence of 8 µg/mL polybrene (Sigma-Aldrich) for 2 h at 37°C. 16 h later, cells were fixed and permeabilized following incubation with 1∶1 methanol-acetone for 10′ at −20°C. To reduce non-specific staining, slides were incubated in blocking reagent (10% normal goat serum, 3% bovine serum albumin, and 1% glycine) for 30′. To identify expression of the latency-associated nuclear antigen (LANA) of KSHV, cells were subsequently incubated with 1∶1000 dilution of an anti-LANA rat monoclonal antibody (ABI) for 1 h, followed by a goat anti-rat secondary antibody (1∶100) conjugated to Texas Red (Invitrogen) for 1 h at 25°C. Nuclei were subsequently counterstained with 0.5 µg/mL 4′,6-diamidino-2-phenylindole (DAPI; Sigma-Aldrich) in 180 mM Tris-HCl (pH 7.5). Slides were examined at 60× magnification using a Nikon TE2000-E fluorescence microscope. Infection rates were determined following examination of at least 200 cells from within 5∼6 random fields in each group. For RAW cell experiments, comparisons between groups are reported as relative infections rates, where relative infection rate = # infected cells per 200 cells in experimental group/infected cells per 200 cells in control group. For RAW cell experiments, comparisons between groups are reported as relative infections rates, where relative infection rate = # infected cells per 200 cells in experimental group/# infected cells per 200 cells in control group. Since HUVEC are more permissive for infection and the majority of cells exhibit at least 1–2 LANA dots (episomes) at MOI∼1, we calculated relative LANA expression for HUVEC experiments as follows: relative LANA expression = # LANA dots per 200 cells in experimental group/# LANA dots per 200 cells in control group.

### Biochemical assays

1×10^4^ RAW cells or HUVEC were seeded per well in eight-well chamber slides and incubated with 10 mM Msg, 0.3 mM Sul or 0.01–1.0 mM L-NMMA for 12 h at 37°C, then incubated with cell-free KSHV for 2 h at 37°C. After 12 h, LANA expression within RAW cells was determined by IFA as outlined above.

### Toll-like receptor inhibition

RAW cells were transfected with 1 µg of pcDNA-miRNA or empty vector control, and after 24 h, incubated with 10 mM of either drug vehicle or a double-stranded RNA-activated protein kinase (PKR) inhibitor 2-Aminopurine (InvivoGen, San Diego, CA) for an additional 3 h. In parallel experiments, transfectants were incubated with 100 µM of MyD88 inhibitory peptide or control peptide (Imgenex, San Diego, CA) for an additional 24 h. Nitrite concentration was quantified within culture supernatants as detailed previously.

### Cell viability assays

Cell viability was assessed using a standard MTT assay as previously described [70]. A total of 5×10^3^ RAW cells were incubated in individual wells in a 96-well plate for 24 h. Serial dilutions of L-NMMA were then added and after 24–48 h, cells were incubated in 1 mg/ml of MTT solution (Sigma-Aldrich) at 37°C for 3 h then 50% DMSO overnight and optical density at 570 nm determined by spectrophotometer (Thermo Labsystems). For assessing cell viability in an environment of oxidative stress, we transfected or infected RAW cells with pcDNA-miRNA (pcDNA control) or KSHV (or UV-KSHV control) in the presence of siRNA targeting xCT or control non-target siRNA. Thereafter, cells were incubated for 12 h with SNAP and cell viability determined using 0.4% trypan blue (MP Biomedicals, Solon, Ohio) to identify dead cells under light microscopy. Relative differences for cell viability between groups was determined as follows: relative cell viability = dead cells per 200 cells in experimental group/dead cells per 200 cells in vehicle control group.

### PCR

Total DNA was isolated using the QIAamp DNA Mini kit (QIAGEN). Briefly, cells were trypsinized for 5′ at 37°C and collected with 1 mL of ice-cold DMEM. Cells were pelleted at 2,000 rpm for 5′, washed, and resuspended in 200 µL of 1-phosphate-buffered saline (PBS), and total DNA was prepared according to the manufacturer's instructions. Total RNA was isolated using the RNeasy Mini kit (QIAGEN) as previously demonstrated [41]. cDNA was synthesized from total RNA using SuperScript III First-Strand Synthesis SuperMix Kit (Invitrogen) according to the manufacturer's instructions. Coding sequences for genes of interest and β-actin (loading control) were amplified from 200 ng input cDNA and using iQ SYBR Green Supermix (Bio-rad). Custom primers sequences used for amplification experiments (Operon) were as follows: *LANA sense 5′ TCCCTCTACACTAAACCCAATA 3′; LANA antisense 5′ TTGCTAATCTCGTTGTCCC 3′; BACH-1 sense 5′ AGGACCTCACGGGCTCTA 3′; BACH-1 antisense 5′ ACCCAACCAGGGACACTC 3′; xCT sense 5′ GGTGGAACTGCTCGTAAT 3′; xCT antisense 5′ CAAAGATCGGGACTGCTA 3′; β-actin sense 5′ GGGAATGGGTCAGAAGGACT 3′; β-actin antisense 5′ TTTGATGTCACGCACGATTT 3′*. Amplification experiments were carried out on an iCycler IQ Real-Time PCR Detection System, and cycle threshold (Ct) values tabulated in triplicate (DNA) or duplicate (cDNA) for each gene of interest for each experiment. “No template” (water) controls were also used to ensure minimal background contamination. Using mean Ct values tabulated for different experiments and Ct values for β-actin as loading controls, fold changes for experimental groups relative to assigned controls were calculated using automated iQ5 2.0 software (Bio-rad). Target amplification for semi-quantitative PCR (RT-PCR) was performed using a DNA thermal cycler (Gene Amp PCR System 9700, Applied Biosystems) under conditions of 94°C for 5′, 35 cycles of 94°C for 30 s, 54°C for 30 s, and 72°C for 60 s. Amplicons were subsequently identified by ethidium bromide-loaded agarose gel electrophoresis.

### Immunohistochemistry

Archived, paraffin-embedded KS skin lesions were collected from the Medical University of South Carolina (MUSC) Hollings Cancer Center Tumor Bank and the Maize Center for Dermatopathology (Charleston, S.C.). The diagnosis of KS and the histopathologic stage of each lesion were verified by an independent dermatopathologist. Histopathologic staging was determined using published criteria [71] to characterize lesions as patches, plaques or nodules (with nodules representing the most advanced lesional stage). Tissue sections were deparaffinized and hydrated through xylene and graded alcohol series, rinsed for 5′ in distilled water, incubated for 10′ in 3% hydrogen peroxide, and following PBS wash, incubated for 30′ in a commercial antigen retrieval solution (Vector Laboratories, Burlingame, CA) at 100°C. Thereafter, all sections were incubated in 100% rabbit serum for 30′ to reduce non-specific staining then for 1 hour with either control preimmune rabbit sera or xCT antisera diluted 1∶400. In parallel, representative sections were incubated with 1∶1200 dilution of the anti-LANA rat monoclonal antibody (ABI). All sections were subsequently incubated for 30′ with a commercially available biotinylated secondary antibody and reagents according to the manufacturer's instructions (Vector Laboratories, Burlingame, CA). Bound antibodies were recognized using a 3,3′- Diaminobenzidine (DAB) substrate and nuclei identified using either hematoxylin to contrast xCT expression, or Methyl Green to contrast LANA expression. xCT expression was determined for at least 8 independent tumors representing each of three histopathologic stages of KS (patches, plaques, and nodules). LANA expression was determined for patch and nodular lesions in this cohort.

### Statistical analysis

Significance for differences between experimental and control groups was determined using the two-tailed Student's t-test (Excel 8.0), and p values <0.05 or <0.01 were considered significant or highly significant, respectively.

## Supporting Information

Figure S1 Upregulation of xCT does not increase the total number of infected HUVEC. (A) HUVEC were incubated with vehicle (DMSO), Msg or Sul for 12 h followed by purified KSHV at MOI∼0.5–1 for which a fraction (approximately 20%) of control HUVEC exhibited no LANA expression 16 h later by IFA. (B) HUVEC were transfected with either control vector or miRNA-expressing vectors along with an inhibitor of miR-K12-11 or either control non-target siRNA (n) or xCT-specific siRNA prior to their incubation with KSHV. Relative infection rates were determined for all groups as previously described. Error bars represent the S.E.M. for three independent experiments.
(0.07 MB TIF)

Figure S2 KSHV miRNAs induce RNS release by macrophages independent of toll-like receptor pathway activation. (A) RAW cells were transiently transfected with either control or miRNA-expressing vectors for 24 h, then incubated with 10 mM 2-aminopurine (2-AP) or vehicle control for 3 h. (B) In parallel, RAW cells were transfected as in (A), then incubated with 100 µM of a control peptide or MyD88 inhibitor peptide for 24 h prior to nitrite quantification within culture supernatants. Error bars represent the S.E.M. for three independent experiments. * = p<0.05.
(0.08 MB TIF)

Figure S3 L-NMMA induces no discernable toxicity for RAW cells. RAW cells were incubated with the indicated concentrations of L-NMMA and cell viability determined after 48 h by standard MTT assay according to the manufacturer's instructions. Error bars represent the S.E.M. for three independent experiments.
(0.09 MB TIF)

Figure S4 Advanced KS lesions contain more KSHV-infected cells relative to early-stage lesions. Representative early (I) and late (III) stage lesions were processed for immunohistochemistry as described in Methods using secondary antibodies alone (control) or anti-LANA antibodies followed by secondary antibodies (LANA). LANA expression is indicated by dark brown, punctate intranuclear staining seen best at higher power (representative LANA^+^ cells are identified with red arrows). Images are shown at original magnification ×20 or 50.
(1.60 MB TIF)
