# Supplementary figures and images for: Upregulation of xCT by KSHV-Encoded microRNAs Facilitates KSHV Dissemination and Persistence in an Environment of Oxidative Stress
Source: PLoS Pathog. 2010 Jan 29;6(1):e1000742. doi: 10.1371/journal.ppat.1000742 (PMC2813276; doi:10.1371/journal.ppat.1000742)

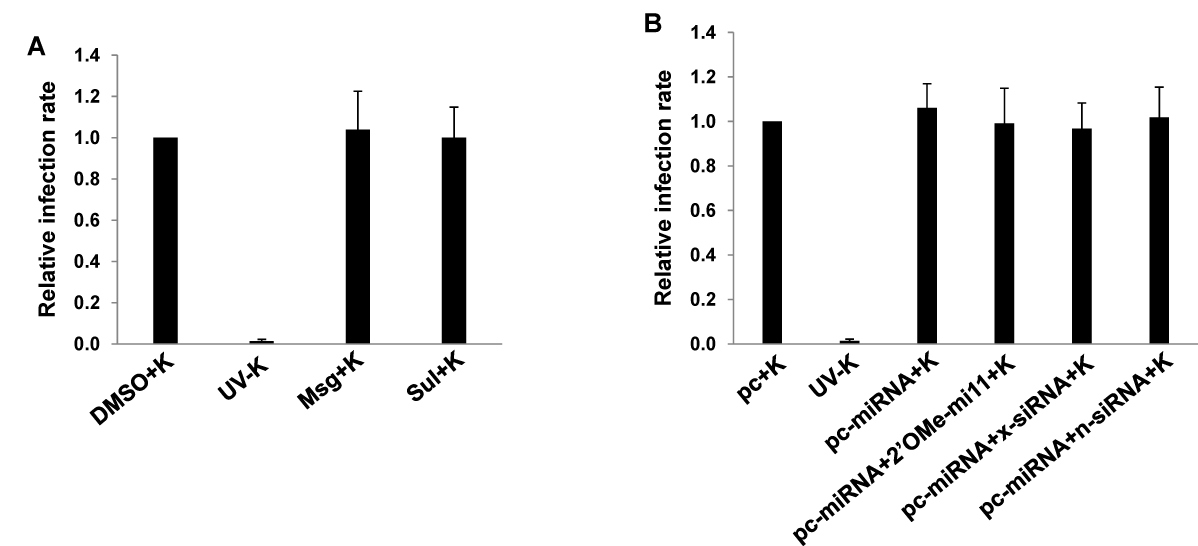

Supplement: Figure S1 — Upregulation of xCT does not increase the total number of infected HUVEC. (A) HUVEC were incubated with vehicle (DMSO), Msg or Sul for 12 h followed by purified KSHV at MOI∼0.5–1 for which a fraction (approximately 20%) of control HUVEC exhibited no LANA expression 16 h later by IFA. (B) HUVEC were transfected with either control vector or miRNA-expressing vectors along with an inhibitor of miR-K12-11 or either control non-target siRNA (n) or xCT-specific siRNA prior to their incubation with KSHV. Relative infection rates were determined for all groups as previously described. Error bars represent the S.E.M. for three independent experiments. (0.07 MB TIF) [file ppat.1000742.s001.tif]

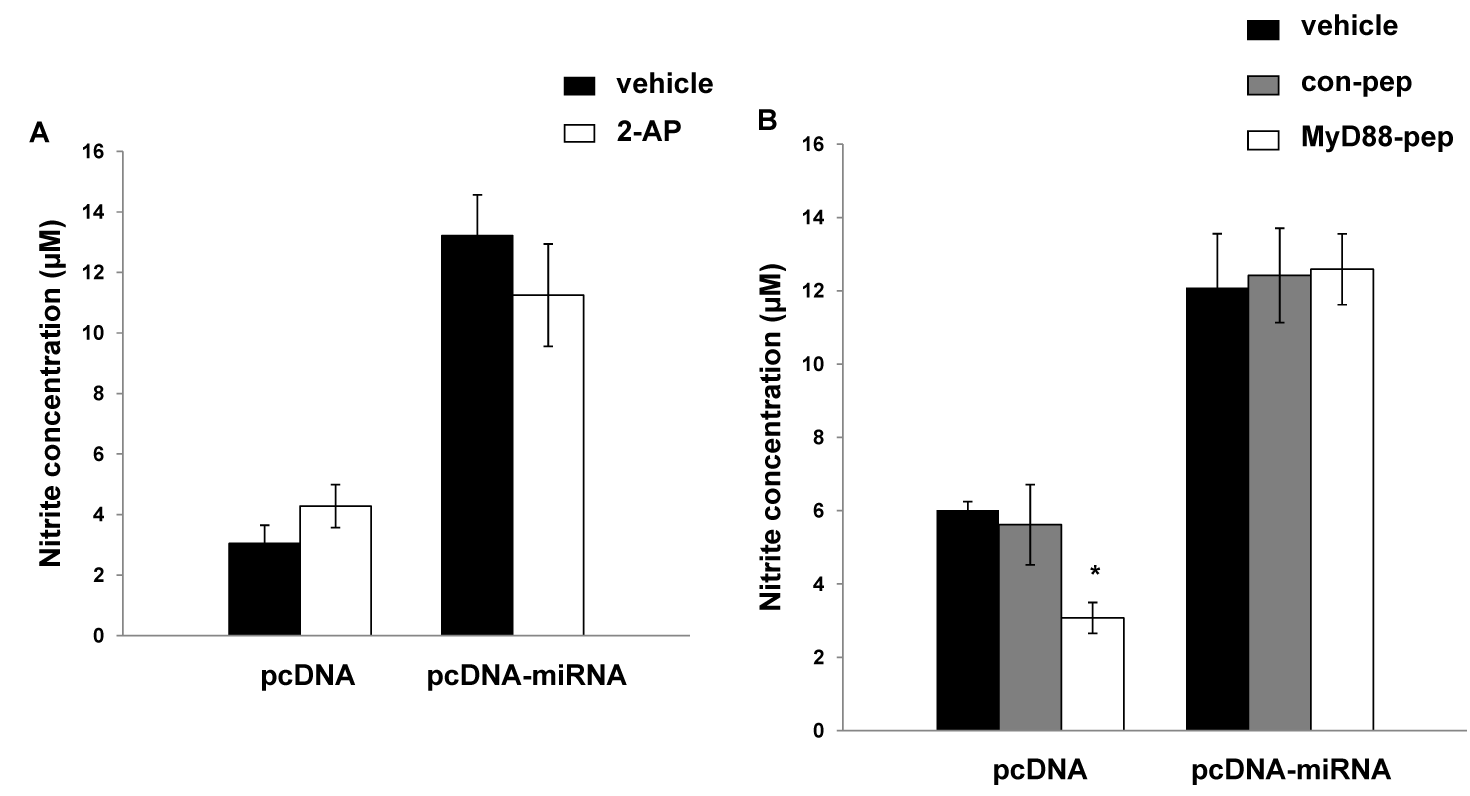

Supplement: Figure S2 — KSHV miRNAs induce RNS release by macrophages independent of toll-like receptor pathway activation. (A) RAW cells were transiently transfected with either control or miRNA-expressing vectors for 24 h, then incubated with 10 mM 2-aminopurine (2-AP) or vehicle control for 3 h. (B) In parallel, RAW cells were transfected as in (A), then incubated with 100 µM of a control peptide or MyD88 inhibitor peptide for 24 h prior to nitrite quantification within culture supernatants. Error bars represent the S.E.M. for three independent experiments. * = p<0.05. (0.08 MB TIF) [file ppat.1000742.s002.tif]

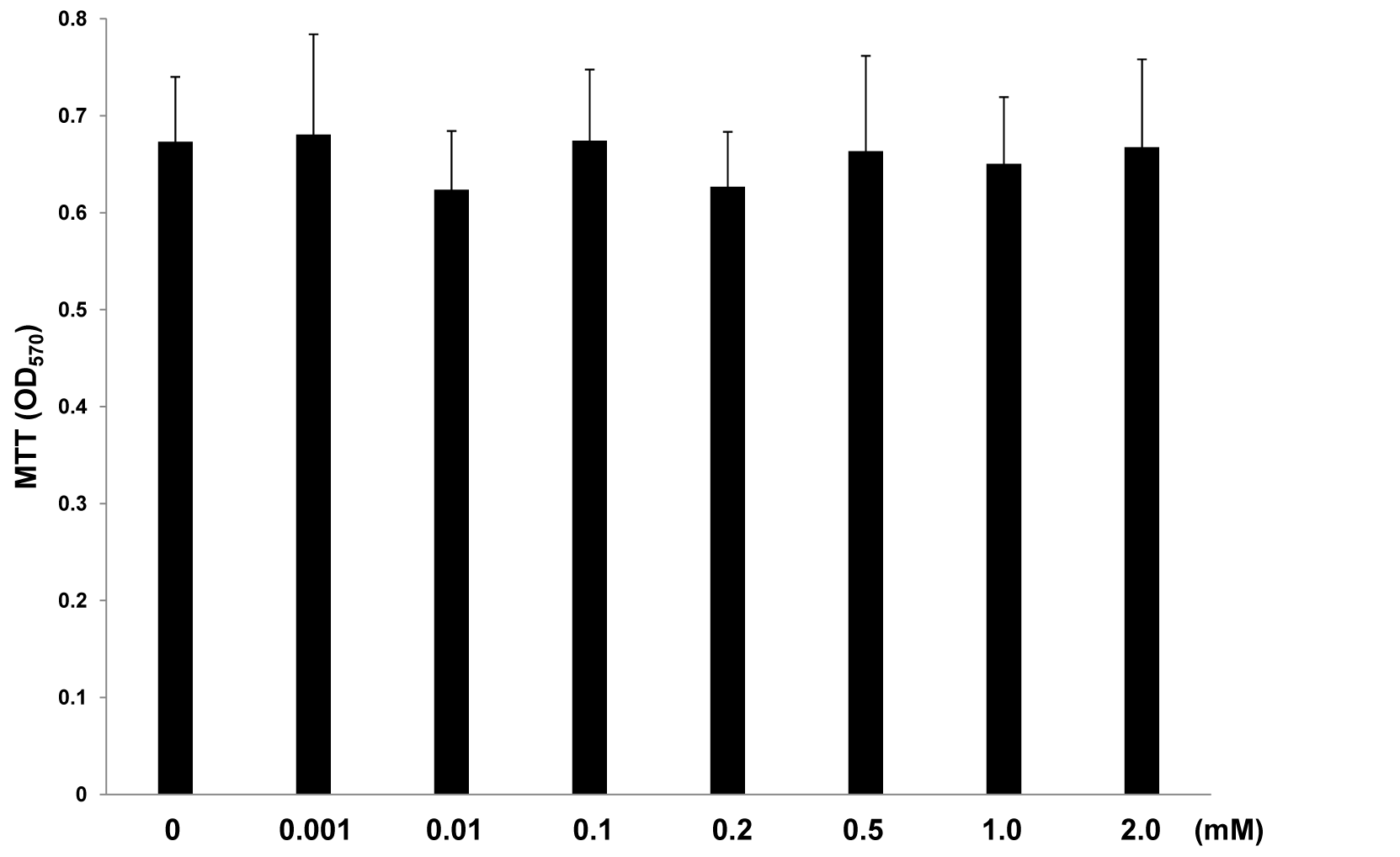

Supplement: Figure S3 — L-NMMA induces no discernable toxicity for RAW cells. RAW cells were incubated with the indicated concentrations of L-NMMA and cell viability determined after 48 h by standard MTT assay according to the manufacturer's instructions. Error bars represent the S.E.M. for three independent experiments. (0.09 MB TIF) [file ppat.1000742.s003.tif]

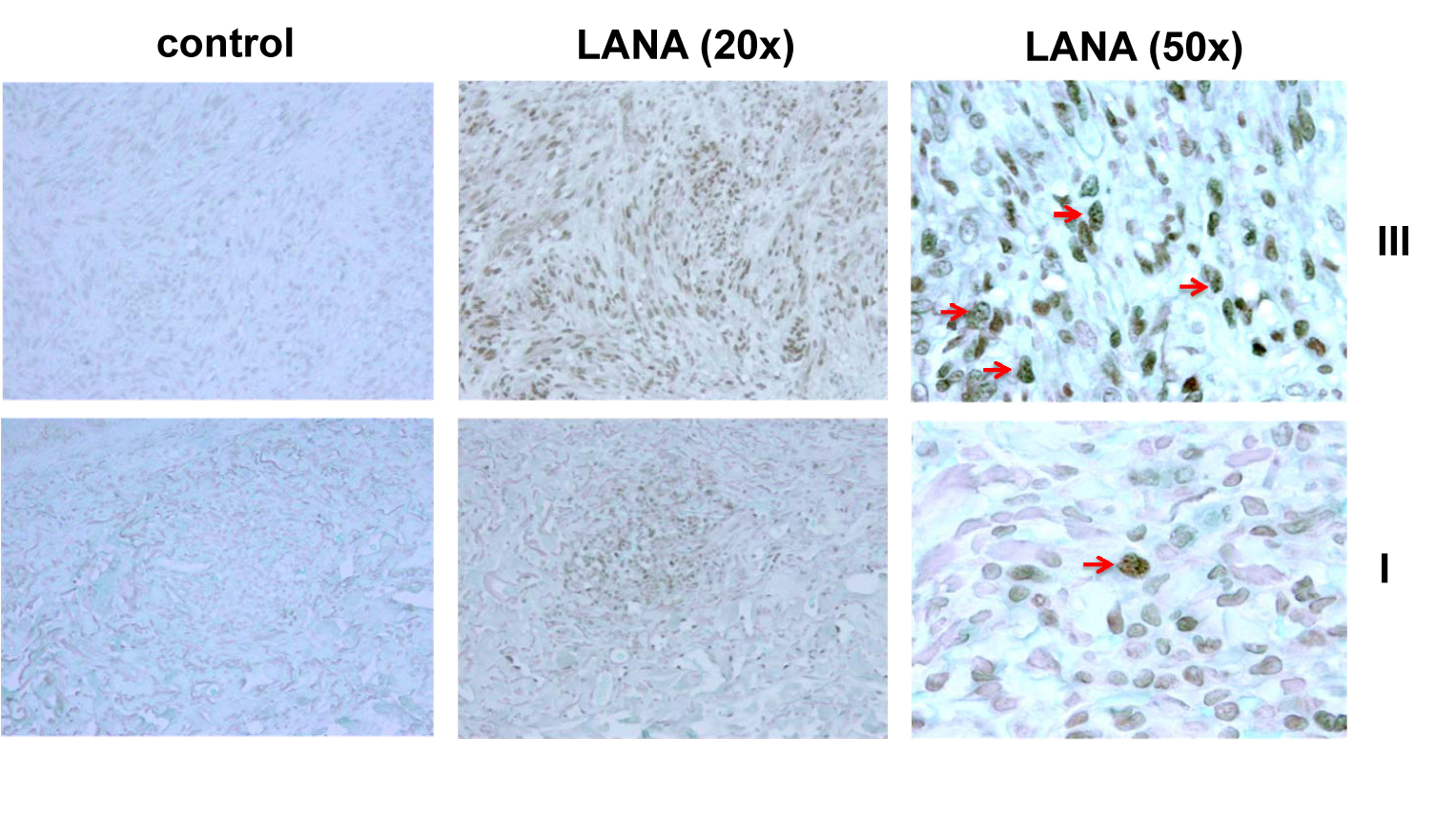

Supplement: Figure S4 — Advanced KS lesions contain more KSHV-infected cells relative to early-stage lesions. Representative early (I) and late (III) stage lesions were processed for immunohistochemistry as described in Methods using secondary antibodies alone (control) or anti-LANA antibodies followed by secondary antibodies (LANA). LANA expression is indicated by dark brown, punctate intranuclear staining seen best at higher power (representative LANA+ cells are identified with red arrows). Images are shown at original magnification ×20 or 50. (1.60 MB TIF) [file ppat.1000742.s004.tif]
